# Genome-wide identification of sucrose nonfermenting-1-related protein kinase (SnRK) genes in barley and RNA-seq analyses of their expression in response to abscisic acid treatment

**DOI:** 10.1186/s12864-021-07601-6

**Published:** 2021-04-26

**Authors:** Zhiwei Chen, Longhua Zhou, Panpan Jiang, Ruiju Lu, Nigel G. Halford, Chenghong Liu

**Affiliations:** 1 Biotechnology Research Institute, Shanghai Academy of Agricultural Sciences, Shanghai, 201106 China; 2 Shanghai Key Laboratory of Agricultural Genetics and Breeding, Shanghai, 201106 China; 3 Shenzhen RealOm ics (Biotech) Co., Ltd., Shenzhen, 518081 China; 4 Plant Sciences Department, Rothamsted Research, Harpenden, Hertfordshire, AL5 2JQ UK

**Keywords:** Barley, *Hordeum vulgare*, SnRK, Sucrose nonfermenting-1, Gene family, Abscisic acid, Metabolic regulation, Stress responses

## Abstract

**Background:**

Sucrose nonfermenting-1 (SNF1)-related protein kinases (SnRKs) play important roles in regulating metabolism and stress responses in plants, providing a conduit for crosstalk between metabolic and stress signalling, in some cases involving the stress hormone, abscisic acid (ABA). The burgeoning and divergence of the plant gene family has led to the evolution of three subfamilies, *SnRK1*, *SnRK2* and *SnRK3*, of which *SnRK2* and *SnRK3* are unique to plants. Therefore, the study of SnRKs in crops may lead to the development of strategies for breeding crop varieties that are more resilient under stress conditions. In the present study, we describe the *SnRK* gene family of barley (*Hordeum vulgare*), the widespread cultivation of which can be attributed to its good adaptation to different environments.

**Results:**

The barley *HvSnRK* gene family was elucidated in its entirety from publicly-available genome data and found to comprise 50 genes. Phylogenetic analyses assigned six of the genes to the *HvSnRK1* subfamily, 10 to *HvSnRK2* and 34 to *HvSnRK3*. The search was validated by applying it to Arabidopsis (*Arabidopsis thaliana*) and rice (*Oryza sativa*) genome data, identifying 50 *SnRK* genes in rice (four *OsSnRK1*, 11 *OsSnRK2* and 35 *OsSnRK3*) and 39 in Arabidopsis (three *AtSnRK1*, 10 *AtSnRK2* and 26 *AtSnRK3*). Specific motifs were identified in the encoded barley proteins, and multiple putative regulatory elements were found in the gene promoters, with light-regulated elements (LRE), ABA response elements (ABRE) and methyl jasmonate response elements (MeJa) the most common. RNA-seq analysis showed that many of the *HvSnRK* genes responded to ABA, some positively, some negatively and some with complex time-dependent responses.

**Conclusions:**

The barley *HvSnRK* gene family is large, comprising 50 members, subdivided into *HvSnRK1* (6 members), *HvSnRK2* (10 members) and *HvSnRK3* (34 members), showing differential positive and negative responses to ABA.

**Supplementary Information:**

The online version contains supplementary material available at 10.1186/s12864-021-07601-6.

## Background

Sucrose nonfermenting-1 (SNF1)-related protein kinases (SnRKs) are related to SNF1 of fungi and AMP-activated protein kinase (AMPK) of mammals (see [1] for review). These protein kinases play important roles in regulating metabolism in all three systems, but in plants the SnRK family has expanded and diverged into three subfamilies, SnRK1 (structurally and functionally the most similar to SNF1 and AMPK), SnRK2 and SnRK3 [2]. SnRKs have been shown to be involved not only in the regulation of metabolism in plants but also in stress responses (see [2, 3] for reviews). Indeed, the burgeoning and divergence of this family of protein kinases in plants may have occurred to allow cross-talk between metabolic and stress signalling, enabling plants to use metabolic changes to adapt to stress conditions, for example by interchanging simple sugars and polysaccharides [3]. Therefore, the study of SnRKs in crops may lead to the development of strategies for breeding crop varieties that are more resilient under stress conditions. This is particularly important in the face of climate change and the prediction that extreme weather events are likely to become more frequent and more severe in the coming decades, with the potential for serious impacts on crop yield and quality.

A *SnRK1* cDNA, initially called *RKIN1*, was first cloned from a rye (*Secale cereale*) endosperm cDNA library and shown to restore SNF1 function in yeast (*Saccharomyces cerevisiae*) *snf1* mutants [4]. In fact, the gene usually referred to as *SnRK1* in plants actually encodes a catalytic subunit of a heterotrimeric protein. AMPK also comprises three subunits, a catalytic α subunit and accessory β and γ subunits, while the yeast protein comprises SNF1 (equivalent to AMPKα), SNF4 (equivalent to AMPKγ) and one of three related proteins, SIP1, SIP2 and GAL83 (related to AMPKβ) [2]. For this reason, the *SnRK1* gene is sometimes referred to as *AMPKα*.

SnRK1 controls metabolism in part through the phosphorylation of enzymes such as 3-hydroxy-3-methylglutaryl coenzyme A reductase (HMG-CoA reductase) and sucrose phosphate synthase, leading to their inactivation. It also phosphorylates nitrate reductase, trehalose-phosphate synthase, and 6-phosphofructo-2-kinase/fructose-2,6-bisphosphatase, but these enzymes also require the binding of a 14–3-3 protein for inactivation (see [2] for review). Another important metabolic enzyme, adenosine diphosphate (ADP)-glucose pyrophosphorylase, is regulated by SnRK1 through modulation of its redox state [5]. SnRK1 also acts through the regulation of gene activity, causing changes in gene expression in response to nutrient starvation [6] and herbivory [7]. SnRK1 has also been shown to channel carbon into the starch biosynthetic pathway in potato (*Solanum tuberosum*) tubers through modulation of sucrose synthase and ADP-glucose pyrophosphorylase gene expression [8, 9], and there is evidence that it plays a similar role in rice (*Oryza sativa*), sorghum (*Sorghum bicolor*) and maize (*Zea mays*) grain [10, 11] as well as sorghum and barley (*Hordeum vulgare*) pollen [11, 12]. Interestingly, both SNF1 and AMPK are required for storage carbohydrate synthesis in their respective systems, although the storage carbohydrate is different (glycogen instead of starch), the glucose donor is different (UDP-glucose instead of ADP-glucose) and the mechanisms of regulation are different [13, 14]. Paradoxically, SnRK1 is also required for the expression of *a-Amy2* (α-amylase), a sugar-repressed gene that is involved in starch breakdown during germination [15].

The fact that SnRK1 phosphorylates and inactivates nitrate reductase provides a route through which SnRK1 can affect nitrogen metabolism. A second route is via asparagine synthetase gene expression, which has been shown to be regulated by SnRK1 in Arabidopsis (*Arabidopsis thaliana*) [6]. In wheat (*Triticum aestivum*), asparagine synthetase gene expression in the developing embryo increases in response to sulphur deficiency, and SnRK1 has been implicated in regulating this response [16, 17]. Cereals contain a class of *SnRK1* genes that are expressed endosperm-specifically [18, 19] and these have been called *SnRK1b* [1]. A third class that is closely related to *SnRK1b* but is expressed in the embryo as well as the endosperm has recently been identified and called SnRK1b* [16]. It was this class that was suggested to be regulating asparagine synthetase gene expression in the wheat embryo [16].

There is also growing evidence that SnRK1 plays an important role in the defence against pathogens (see [20, 21] for reviews). Over-expression of *SnRK1* in tomato (*Solanum lycopersicum*), for example, has been found to enhance viral resistance, while antisense suppression has the opposite effect [22]. *SnRK1* overexpression has also been shown to confer broad-spectrum disease resistance in rice [23, 24], enhancing the defence response mediated by jasmonate [24]. In wheat, SnRK1 has been shown to interact with a protein, TaFROG, that enhances wheat’s resistance to deoxynivalenol (DON), a mycotoxin produced by pathogenic *Fusarium* fungi [25]. DON treatment increases SnRK1 phosphorylation/activation and activity, while down-regulation of *SnRK1* gene expression using virus-induced gene silencing (VIGS) increases the damaging effects of DON on wheat spikelets [25].

SnRK1 will phosphorylate peptides containing a serine residue (much preferred to threonine) with hydrophobic residues at positions − 5 and + 4 with respect to the serine, and a basic residue at position − 3 or, less favourably, − 4 [1, 26]. However, plant extracts generally contain two additional activity peaks that will phosphorylate this target site, as well as the one accounted for by SnRK1 [26–29], and these came to be called SnRK2 and SnRK3 [1]. It was soon apparent that a *SnRK2* cDNA had already been cloned from wheat and named *pkABA1*, the name being assigned because *pkABA1* mediates ABA-induced changes in gene expression in response to cold, dehydration and osmotic stress [30, 31]. A very similar *SnRK2* gene, *SAPK2*, confers ABA sensitivity and drought tolerance in rice [32]. The *SnRK2* gene family is unique to plants and has expanded and diversified to comprise, for example, 10 members in Arabidopsis [2]. SnRK2s are much smaller than SnRK1, with molecular weights typically around 40 kDa compared with 58 kDa for SnRK1, and do not complement the *snf1* mutation of yeast [1]. Indeed, SnRK2s are only large enough to include a protein kinase domain and a short, truncated, acidic C-terminal domain. They are associated with a variety of abiotic stress responses [3, 33–36], and are integral to the abscisic acid (ABA) response pathway [34, 37–39], becoming active in response to ABA and phosphorylating transcription factors of the ABA-response-element-binding protein class (AREBPs). These transcription factors have been shown to be substrates for SnRK1 and SnRK3 as well [40], making them potential convergence points for multiple signalling pathways.

Halford and Hardie [1] divided SnRK2s into two subclasses, SnRK2a and SnRK2b, based on whether their acidic C-terminal domain contained an aspartic acid- or glutamic acid-rich patch. Subsequently, Kobayashi et al. [33] identified three subclasses, I, II and III, with subclass I equivalent to SnRK2b (glutamic acid-rich C-terminal patch) and subclasses II and III representing subdivisions of SnRK2a (aspartic acid-rich patch). These subclasses have been shown to respond differently to ABA, with subclass I not activated by ABA, subclass II not activated or activated very weakly by ABA, and subclass III strongly activated by ABA [41–44]. ABA is, of course, strongly associated with stress responses (see [45] for review), but it is also a key player in driving grain maturation and, in particular, the switch from the grain filling to maturation phases of grain development [46]. ABA has a very different effect on SnRK1 than SnRK2, promoting SnRK1 degradation in wheat roots, for example, while promoting SnRK2 phosphorylation/activation [47]. It has been suggested that the ABA-driven degradation of SnRK1 together with the activation of SnRK2 could be the trigger that pushes developing grain into the maturation phase [48].

The first *SnRK3* gene to be characterized was also from wheat, and was initially named *wpk4* [49, 50]. The *SnRK3* gene family is even larger than the *SnRK2* family, with the Arabidopsis genome containing 25 *SnRK3* genes [2]. Unlike SnRK1 and SnRK2, SnRK3 is believed to be calcium-dependent, because it interacts with a calcium-binding protein, calcineurin B-like protein (CBL) [36, 51–53]. This has led to SnRK3s being more popularly known as CIPKs (CBL-interacting protein kinases). One of the most extensively characterized SnRK3/CIPKs is *SOS2* (salt overly sensitive 2), which is involved in the response to salt stress [51, 54–57]. Sodium ions are just one of many species of ions that plants take up from the soil, of course, and, while many are important nutrients, some can be toxic at high concentrations. SnRK3s/CIPKs are now considered to be involved generally in enabling plants to adapt to changing ionic conditions. Their roles have recently been reviewed in depth by Tang et al. [58] and Ma et al. [59].

SnRKs represent one of the largest families within the plant kinome, and their positioning at the interface between metabolic, stress and ABA signalling has made them the focus of studies in many plant species. As whole genome data have become available for many species, along with powerful bioinformatics tools, the identification of entire *SnRK* gene familes at the genome level has become possible, for example in Arabidopsis, *Brachypodium distachyon*, Chinese cabbage (*Brassica rapa*), soybean (*Glycine max*), and oilseed rape (*Brassica napus*) [36, 60–63]. However, the role of ABA in driving developmental changes in cereal seed development, the differential effects of ABA on the different SnRK families, and the role of SnRKs in regulating processes that affect grain composition, such as starch accumulation and asparagine biosynthesis, make the study of SnRKs in cereals particularly important. Barley was the first plant species in which a characterization of the *SnRK1* gene family was begun, with three different members of the family, called *BKIN2*, *BKIN9* and *BKIN12* at that time, shown to have different gene structures and to be differentially-expressed, with *BKIN12,* subsequently assigned to the *SnRK1b* class, expressed specifically in the endosperm tissue of the grain [18, 19]. Here we report the analysis of the entire *SnRK* gene family of barley by using the barley genome data from Ensembl. It is the first comprehensive analysis of the *SnRK* gene family of a commercial cereal species.

## Results

### Genome-wide identification of the *SnRK* gene family members in barley

In total, 50 HvSnRKs were found to be encoded in the barley genome (Table 1), of which six were classed as SnRK1s by Pfams analysis and called HvSnRK1.1 – HvSnRK1.6. Each of these contained a kinase associated domain KA1 (Pfams reference PF02149), and all except one, HvSnRK1.2, also contained an ubiquitin-associated domain (UBA) (PF00627). Thirty-four proteins were assigned to the SnRK3/CIPK group based on the presence of a NAF domain (PF03822), a 24 amino acid domain required for binding to CBLs [64]. The remaining 10 proteins were assigned to the SnRK2 group. The proteins comprised between 232 and 797 amino acid residues and had molecular weights ranging from 25.9 to 89.9 kDa (see Table 1). The smallest of these, one of the SnRK3s/CIPKs, is not large enough to include a complete kinase domain and is unlikely to be functional.

**Table 1:** Characteristics of *HvSnRKs* from barley

Gene name	Gene ID	CDS length (bp)	Peptide length (aa)	pI	MW (KDa)	Localization
HvSnRK1.1	HORVU1Hr1G081310	1563	520	8.7	59.04	–
HvSnRK1.2	HORVU3Hr1G069190	1716	571	9.45	65.01	Chloroplast
HvSnRK1.3	HORVU3Hr1G107970	1647	548	6.68	62.19	–
HvSnRK1.4	HORVU3Hr1G107990	1659	552	7.65	63.28	Mitochondrion
HvSnRK1.5	HORVU3Hr1G108000	1041	346	7.06	39.48	–
HvSnRK1.6	HORVU4Hr1G056610	1545	514	8.58	58.81	–
HvSnRK2.1	HORVU1Hr1G055340	1026	341	5.99	38.53	–
HvSnRK2.2	HORVU2Hr1G029900	1389	462	6.52	51.57	–
HvSnRK2.3	HORVU2Hr1G075470	1074	357	5.54	40.90	–
HvSnRK2.4	HORVU2Hr1G110230	1035	344	5.8	39.11	–
HvSnRK2.5	HORVU2Hr1G125950	1125	374	8.55	41.85	–
HvSnRK2.6	HORVU3Hr1G082690	1146	382	7.72	44.21	Mitochondrion
HvSnRK2.7	HORVU4Hr1G013540	1086	361	4.8	40.65	–
HvSnRK2.8	HORVU5Hr1G018340	1116	371	4.99	41.33	–
HvSnRK2.9	HORVU5Hr1G097630	1101	366	4.86	41.53	–
HvSnRK2.10	HORVU0Hr1G011570	1074	357	4.94	40.15	–
HvSnRK3.1	HORVU1Hr1G017240	1479	492	8.49	54.96	–
HvSnRK3.2	HORVU1Hr1G017380	1488	495	8.44	54.91	–
HvSnRK3.3	HORVU1Hr1G070100	1491	497	9.43	56.43	–
HvSnRK3.4	HORVU1Hr1G076910	1533	510	7.24	57.03	Chloroplast
HvSnRK3.5	HORVU2Hr1G016750	1347	448	8.01	50.74	Mitochondrion
HvSnRK3.6	HORVU2Hr1G018260	1353	450	9.07	50.83	–
HvSnRK3.7	HORVU2Hr1G018340	1512	504	6.86	54.48	–
HvSnRK3.8	HORVU2Hr1G027080	1356	451	7.95	51.13	–
HvSnRK3.9	HORVU2Hr1G031190	699	232	5.37	25.90	–
HvSnRK3.10	HORVU2Hr1G031200	825	274	5.67	30.67	–
HvSnRK3.11	HORVU2Hr1G060350	1386	461	9.15	51.77	–
HvSnRK3.12	HORVU2Hr1G117610	1377	458	8.9	50.78	–
HvSnRK3.13	HORVU3Hr1G025810	1536	511	8.4	56.39	Mitochondrion
HvSnRK3.14	HORVU3Hr1G029350	1692	563	8.88	63.16	–
HvSnRK3.15	HORVU3Hr1G073100	1731	576	8.91	63.97	–
HvSnRK3.16	HORVU3Hr1G073110	1437	478	8.88	53.12	–
HvSnRK3.17	HORVU3Hr1G081420	1530	509	8.85	57.65	Mitochondrion
HvSnRK3.18	HORVU4Hr1G021090	1329	442	9.04	48.16	–
HvSnRK3.19	HORVU4Hr1G022630	1335	444	9.16	50.38	Mitochondrion
HvSnRK3.20	HORVU4Hr1G026300	1323	440	7.18	50.41	Mitochondrion
HvSnRK3.21	HORVU4Hr1G052200	1350	449	8.27	50.98	Mitochondrion
HvSnRK3.22	HORVU4Hr1G083080	1338	445	7.67	49.91	Mitochondrion
HvSnRK3.23	HORVU5Hr1G014200	1302	433	9.51	47.50	–
HvSnRK3.24	HORVU5Hr1G046830	1341	447	9.13	50.43	–
HvSnRK3.25	HORVU5Hr1G046880	1323	440	9.25	49.61	–
HvSnRK3.26	HORVU5Hr1G065350	1341	446	9.17	47.92	–
HvSnRK3.27	HORVU5Hr1G065370	1515	504	8.81	55.59	Chloroplast
HvSnRK3.28	HORVU5Hr1G093660	1296	431	8.82	47.16	–
HvSnRK3.29	HORVU6Hr1G025940	2394	797	6.36	89.93	Secretory pathway
HvSnRK3.30	HORVU6Hr1G030150	1329	442	7.91	49.58	–
HvSnRK3.31	HORVU6Hr1G054210	1314	438	8.61	48.28	–
HvSnRK3.32	HORVU7Hr1G089510	1461	486	8.74	54.14	–
HvSnRK3.33	HORVU7Hr1G090260	1518	505	9.11	56.21	Mitochondrion
HvSnRK3.34	HORVU0Hr1G015380	1398	465	9.03	52.86	–

“-” represents any other location. The localization, pI and MW are just predictions

Within the SnRK1 group, HvSnRK1.3, 1.4, 1.5 and 1.6 were most similar to the SnRK1b-type protein annotated previously as BKIN12, while SnRK1.1 and SnRK1.2 were most similar to the SnRK1a-type protein, BKIN2 [18, 19]. HvSnRK1.3 and HvSnRK1.4 were assigned to SnRK1b, while SnRK1.5 and HvSnRK1.6 were assigned to SnRK1b*.

The isoelectric point of the HvSnRK1s ranged from 6.68 to 9.45, the HvSnRK2s from 4.80 to 8.55, and the HvSnRK3s from 5.37 to 9.51 (Table 1). The subcellular localization of HvSnRK1.2, HvSnRK3.4 and HvSnRK3.27 was predicted to be in the chloroplast (Table 1), while HvSnRK1.4, HvSnRK2.6, HvSnRK3.5, HvSnRK3.13, HvSnRK3.17, HvSnRK3.19, HvSnRK3.20, HvSnRK3.21, HvSnRK3.22 and HvSnRK3.33 were predicted to localize to the mitochondrion, and HvSnRK3.29 to the secretory pathway. The others are likely to be cytosolic. The fact that SnRK1 is targeted to different cellular locations has led to the hypothesis that it mediates energy signaling between different organelles [65].

To validate our screen and to compare and contrast the SnRK families across species, we also identified SnRKs from rice and Arabidopsis. The rice family comprised a total of 50 OsSnRKs, made up of four OsSnRK1s, eleven OsSnRK2s and 35 OsSnRK3s, while the Arabidopsis family comprised 39 AtSnRKs, including three AtSnRK1s, 10 AtSnRK2s and 26 AtSnRK3s (Additional file 1). The Arabidopsis family included one additional SnRK3 compared with those identified by Halford and Hey [2].

### Phylogenetic analyses

**Fig. 1:**
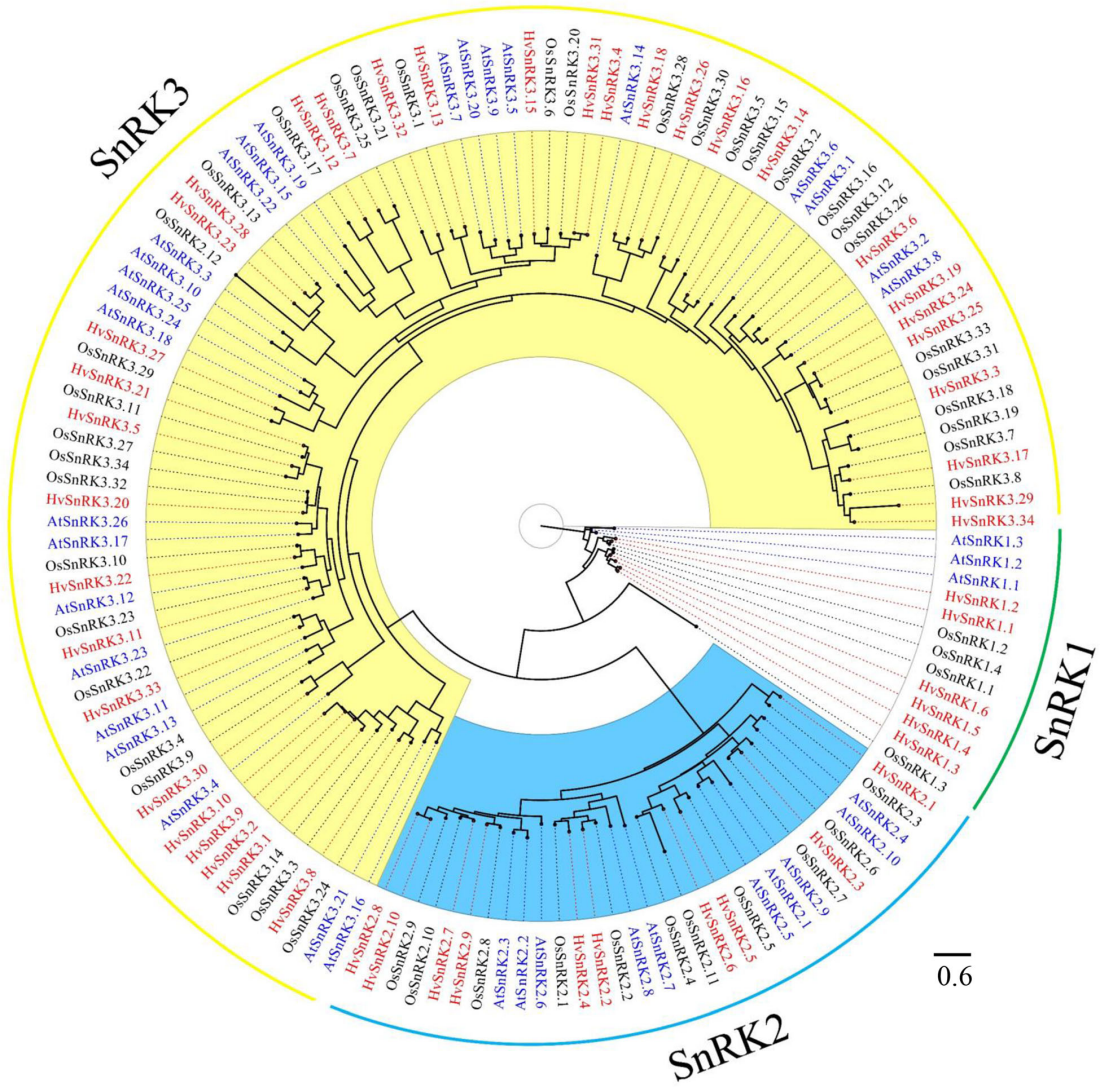
Phylogenetic tree of SnRK proteins from barley, rice and Arabidopsis. The Maximum-Likelihood tree (JTT + I + G + F) was constructed using RAxML with conserved protein kinase domain regions of 139 SnRKs. Different colour fonts represent SnRKs from barley (red), rice (black) and Arabidopsis (blue). Different colour sectors represent SnRKs from SnRK1 subgroup (white), SnRK2 (blue) and SnRK3 (yellow)

A rooted phylogenetic analysis was conducted for all the SnRKs identified from barley, rice and Arabidopsis. The phylogenetic tree that was generated (Fig. 1) confirmed the three major groups of SnRK1, SnRK2 and SnRK3, and the assignation of each barley SnRK to the groups confirmed the Pfams analysis, although one of the rice genes that had been classified as encoding a SnRK2 by Pfams appeared in the SnRK3 cluster in the phylogenetic analysis and was named *OsSnRK3.35*. Note that SnRK1 is more closely related to SNF1 of yeast and AMPK of mammals than it is to SnRK2 and SnRK3 [1]. While SnRK2 and SnRK3 are plant-specific and presumably diverged from SnRK1 after the separation of animals, plants and fungi, they must have evolved rapidly as they took on different functions.

**Fig. 2:**
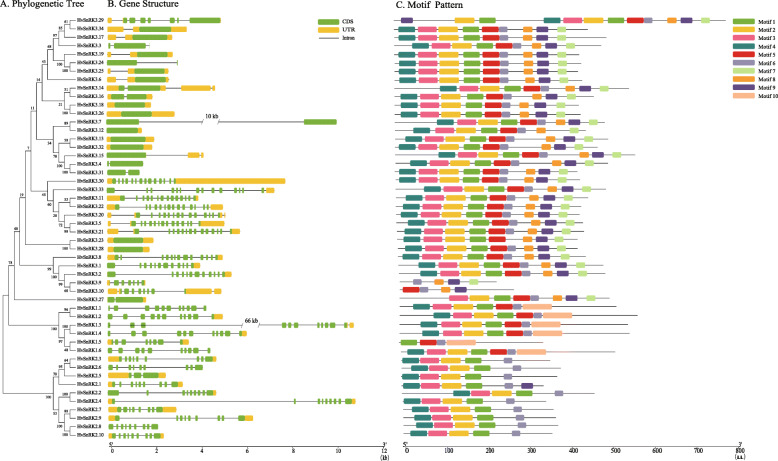
Phylogenetic relationships, gene structure and architecture of conserved protein motifs of the SnRK gene family from barley. **a**. Phylogenetic tree of 50 HvSnRKs constructed using the Neighbor-Joining (NJ) method using MEGA 7.0 with full length amino acids sequences of 50 HvSnRKs proteins. **b**. Gene structures of 50 HvSnRK genes. Green boxes represent exons, yellow boxes represent 5′ or 3′ untranslated regions (UTR), and black lines represent introns. The length of nucleotide sequences of exons/introns/UTRs can be estimated by the scale at the bottom. **c**. The motif compositions of 50 HvSnRK proteins (Figure S2). The motifs were identified using the MEME program. Boxes of different colors represent motif 1 to 10, respectively. The length of amino acid sequences can be estimated by the scale at the bottom

A second phylogenetic tree of the barley HvSnRKs alone was produced using different software. The phylogenetic tree that was generated (Fig. 2a) confirmed the clustering into the HvSnRK1, HvSnRK2 and HvSnRK3 families. As before, the SnRK1 family could be further divided into subclasses, corresponding to SnRK1a (SnRK1.1 and 1.2), SnRK1b (SnRK1.3 and 1.4) and SnRK1b* (SnRK1.5 and 1.6). Similarly, the SnRK2 subgroup was divided into two subclasses, corresponding to SnRK2a and SnRK2b as defined by Halford and Hardie [1] and SnRK2 subclass II/III and SnRK2 subclass I as defined by Kobayashi et al. [33] (subclasses II/III did not separate clearly in this analysis).

### Gene structure, conserved motifs and promoter analysis

There were 11 exons in all the *HvSnRK1* genes except for *HvSnRK1.5*, and 9 exons in all of the *HvSnRK2* genes except for *HvSnRK2.7*. In contrast, the exon/intron structure of the *HvSnRK3s* varied greatly, ranging from 1 exon to 16 (Fig. 2b), indicating that the genes in the *HvSnRK1* and *HvSnRK2* subfamilies are more conserved in structure than those in the *HvSnRK3* subfamily.

The motif analysis of the HvSnRK proteins (Fig. 2c) showed that all the HvSnRK1s had seven motifs, 1, 2, 3, 4, 5, 6 and 10, apart from HvSnRK1.5, which lacked the first three motifs. All of the HvSnRK2s had motifs 1, 2, 3, 4 and 6; in other words they lacked motifs 5 and 10 of the HvSnRK1s. They are, of course, readily distinguishable from the SnRK1s on the basis of size, being much smaller (around 40 kDa compared with around 58 kDa). That is not true of the SnRK3s, which vary considerably in size. However, the SnRK3s were characterized by the presence of the first six motifs identified in the SnRK1s but also by three additional motifs, 7, 8 and 9, that were not present in the SnRK1s, and the lack of motif 10. The exceptions were HvSnRK3.9, which lacked the first five motifs, HvSnRK3.10, which lacked the first four motifs and motif 7, HvSnRK3.27, which lacked the first motif, and HvSnRK3.27, which had additional motifs 1, 2 and 9 before the shared nine motifs. Interestingly, motif 9 was also present in SnRK2.1, but motifs 7 and 8 were not. The presence/absence of these motifs could allow for the simple allocation of SnRKs from other species into the correct subgroup.

**Fig. 3:**
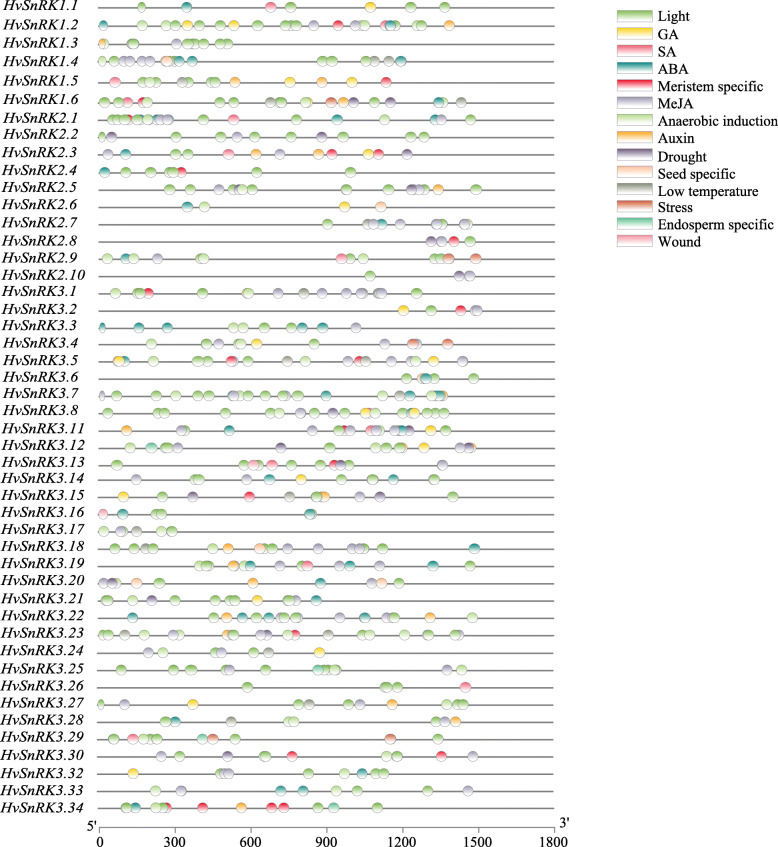
Predicted *cis*-regulatory elements in *HvSnRK* promoters. Promoter sequences (about 1.5 kb) of 50 *HvSnRK* genes were analyzed by PlantCARE (Additional file 1). Boxes (not to scale) represent cis-elements, with different colours representing different types of element. Promoter sequence data were not available for *HvSnRK3.9* or *HvSnRK3.10*, and no *cis*-elements were identified for *HvSnRK3.31*

Considering that HvSnRKs play important roles in many biological processes, the promoters of the genes were analyzed to identify potential regulatory elements that could give clues as to how the genes are regulated and the different stimuli they respond to. Of the 50 *HvSnRK* genes, two (*HvSnRK3.9* and *HvSnRK3.10*) lacked data for the promoter regions, but the analysis identified 18 different types of *cis*-element in the promoter regions of 47 of the other 48 *HvSnRK* genes (Fig. 3, Additional file 2). Apart from the one promoter (that of *HvSnRK3.31*) lacking any of these elements, each promoter contained between 2 and 9 of the different types of *cis*-element, with the promoters of *HvSnRK1.6* and *HvSnRK3.11* containing the most. The most frequently present types of element were light-responsive elements (LRE), abscisic acid (ABA)-responsive elements (ABRE), and methyl jasmonate (MeJA)-responsive elements, which appeared in 46, 39 and 36 of the promoters, respectively. Excepting *HvSnRK3.9*, *HvSnRK3.10* and *HvSnRK3.31*, all of the genes except *HvSnRK2.6* have the LRE. The ABRE is present in the promoters of all the *HvSnRK1s*, all the *HvSnRK2s* apart from *HvSnRK2.8* and *HvSnRK2.10*, and all the *HvSnRK3s* apart from *HvSnRK3.2*, *HvSnRK3.20*, *HvSnRK3.23*, *HvSnRK3.26*, *HvSnRK3.27* and *HvSnRK3.33*. The MeJA-responsive element is present in *HvSnRK1.2*, *HvSnRK1.3* and *HvSnRK1.4*, as well as all the *HvSnRK2s* apart from *HvSnRK2.4* and *HvSnRK2.6*, and all the *HvSnRK3s* apart from *HvSnRK3.1*, *HvSnRK3.6*, *HvSnRK3.16*, *HvSnRK3.26*, *HvSnRK3.29* and *HvSnRK3.34*.

### Chromosomal location, gene duplication and gene synteny analysis

**Fig. 4:**
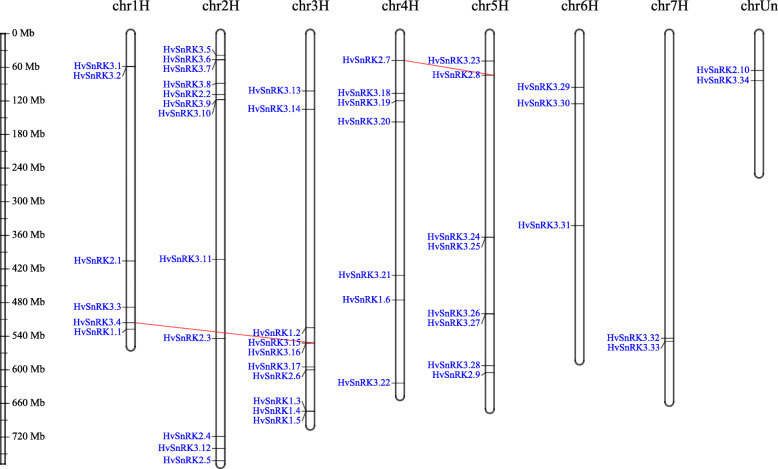
Chromosomal locations of *HvSnRK* genes. Positions linked by red lines represent segmental duplications within *HvSnRK* genes, while yellow rectangles represent tandem duplications with related *HvSnRK* genes. The length of chromosomes can be estimated using the scale on the left. HvSnRK2.10 and HvSnRK3.34 were unclassified

Chromosomal location analysis showed the 50 *HvSnRK* genes to be distributed across all seven barley chromosomes (Fig. 4). Four of the six *HvSnRK1* genes were located to Chromosome 3, with *HvSnRK1.1* on Chromosome 1 and *HvSnRK1.6* on Chromosome 4. *HvSnRK2* genes were distributed on Chromosomes 1–5, with *HvSnRK2.1* on Chromosome 1, *HvSnRK2.2*, *2.3*, *2.4* and *2.5* on Chromosome 2, *SnRK2.6* on Chromosome 3, *SnK2.7* on Chromosome 4, and *HvSnRK2.8* and *2.9* on Chromosome 5. *SnRK2.10* was unclassified. *HvSnRK3* genes were distributed on all of the chromosomes, with most (8) on Chromosome 2.

Gene duplication analysis of the *HvSnRK* genes showed that there were two segmental duplication events between different chromosomes and two tandem duplication events (Fig. 4), while there was only one interval gene in the cluster of *HvSnRK1.3*, *HvSnRK1.4* and *HvSnRK1.5*. The KaKs calculation showed the Ka/Ks values of all the duplication pairs to be less than 1, especially the segmental duplication pairs (Table 2). The results also suggested that the *HvSnRK* genes related to those duplications were conserved, with purifying selection (the selective removal of alleles that are deleterious).

**Table 2:** The KaKs values of the paired duplicated HvSnRK genes

Duplicated genes	Ka	Ks	Ka/Ks
HvSnRK2.7/HvSnRK2.8	0.085723	1.474440	0.058139
HvSnRK3.4/HvSnRK3.15	0.127287	1.294210	0.098351
HvSnRK3.9/HvSnRK3.10	0.011528	0.024877	0.463405
HvSnRK1.3/HvSnRK1.4	0.018936	0.035337	0.535865
HvSnRK1.4/HvSnRK1.5	0.099710	0.245272	0.406528
HvSnRK1.3/HvSnRK1.5	0.096129	0.245078	0.392240

**Fig. 5:**
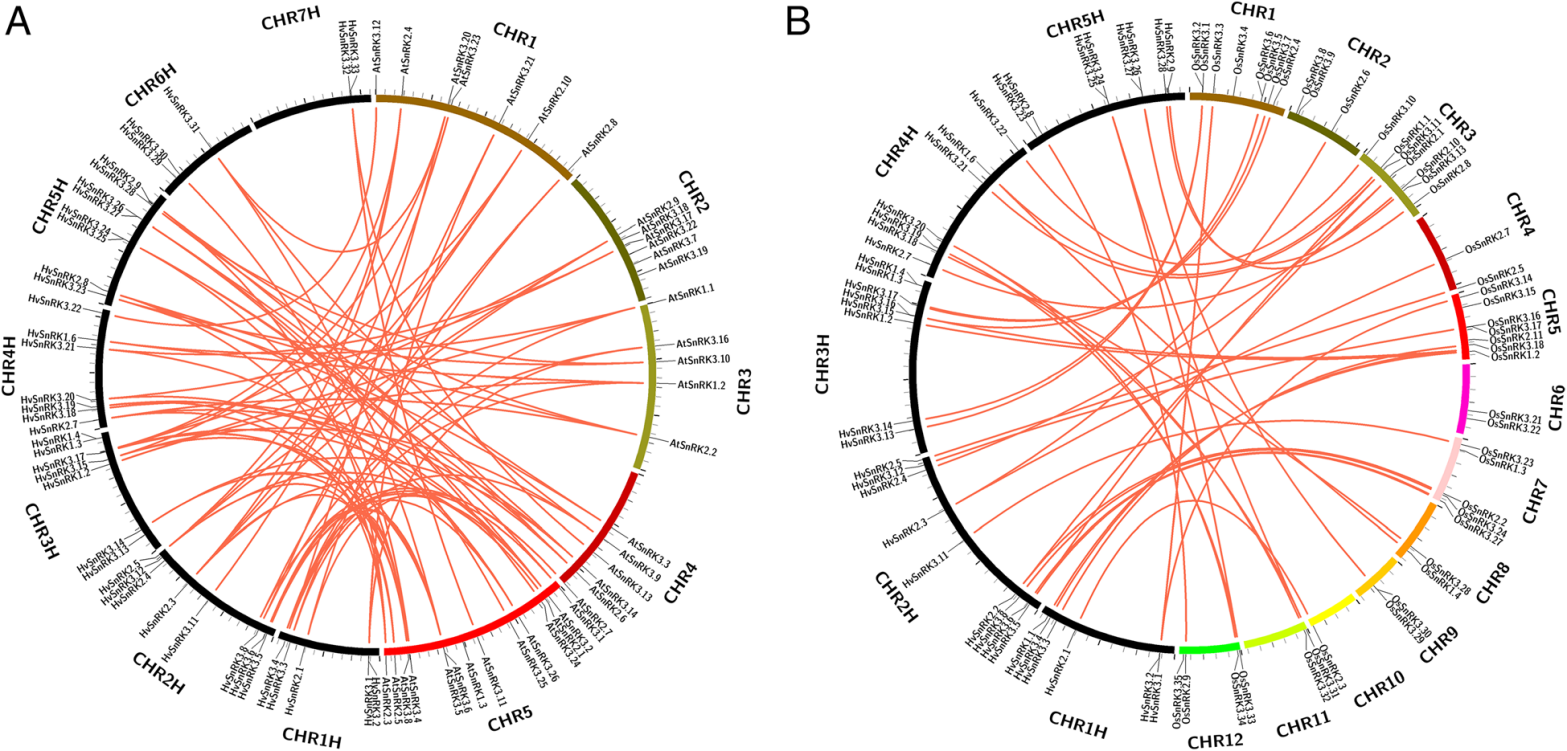
Synteny analyses of *SnRK* genes between **a**. barley and Arabidopsis (Additional file 3. Sheet of synteny analysis 1); **b**. Barley and rice (Additional file 3. Sheet of synteny analysis 2). The genes linked by red lines represent homologues

Gene synteny analyses were performed for the barley *HvSnRK* gene family and the corresponding gene families of Arabidopsis and rice. The results showed that 38 of the 50 *HvSnRKs* had homologues in Arabidopsis and 39 in rice (Fig. 5, Additional file 3). The fact that 11 *HvSnRKs* did not have homologues in either Arabidopsis or rice suggested that the differentiation and specificity of *HvSnRKs* has evolved further in barley. In addition, one *HvSnRK* had several homologues in Arabidopsis, while several *HvSnRKs* were homologous to a single *AtSnRK*. In contrast, most *HvSnRKs* had only one homologous gene in rice.

### Gene expression analysis of *HvSnRKs* in response to ABA treatment

Barley seedlings of variety Morex were cultured in nutrient solution and treated with either 50 μM ABA dissolved in ethanol or ethanol alone (the control). Roots were harvested at 1 h, 3 h, 6 h and 24 h after treatment, with three biological replicates per treatment/time. RNA-seq analysis was performed and reads aligned to the Ensembl Plants database for barley. Gene expression was calculated as the number of reads that mapped to each gene per kilobase of transcript, per million mapped reads (RPKM). The RNA-seq data have been deposited with the National Center for Biotechnology Information: BioProject ID PRJNA661163.

**Fig. 6:**
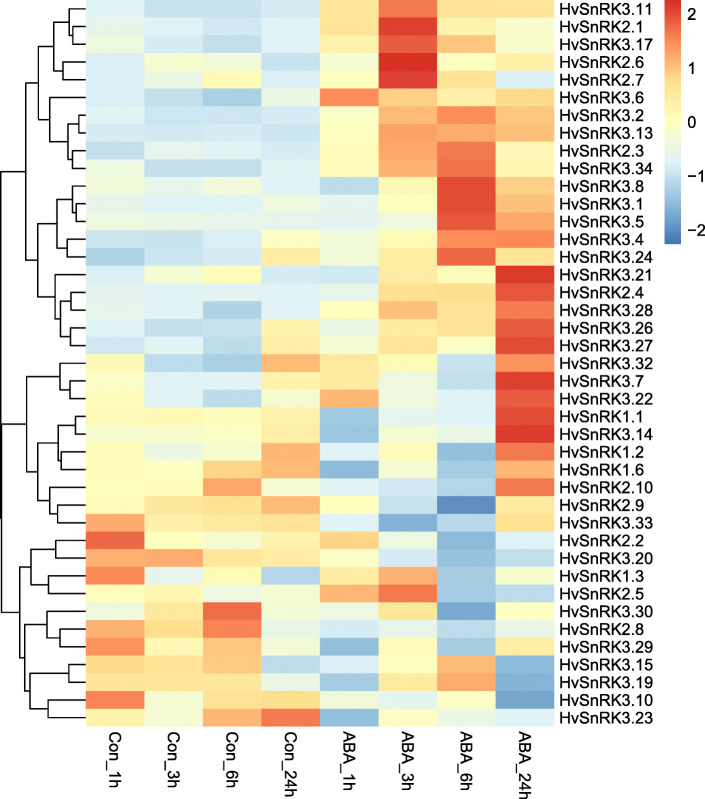
Heatmap showing changes in expression of *HvSnRK* genes in response to ABA treatment in barley roots

In total, 41 of the 50 *HvSnRK* genes were found to be expressed in the roots, and 30 were affected significantly (*p* < 0.05) by the ABA treatment (Fig. 6, Additional file 4). Only one gene, *HvSnRK3.13*, was up-regulated by the ABA treatment at all the time-points, while four genes, *HvSnRK2.4*, *HvSnRK3.1*, *HvSnRK3.2* and *HvSnRK3.9*, were up-regulated at 3 h, 6 h and 24 h. One gene, *HvSnRK3.20*, was down-regulated at 3 h, 6 h and 24 h, while five genes (*HvSnRK3.4*, *HvSnRK3.11*, *HvSnRK3.26*, *HvSnRK3.27* and *HvSnRK3.28*) were up-regulated at 3 h and 6 h of treatment and two genes (*HvSnRK3.5* and *HvSnRK3.8*) were up-regulated at 6 h and 24 h of treatment. *HvSnRK3.19* was up-regulated at 1 h after ABA treatment but not at the other timepoints, while *HvSnRK2.6*, *HvSnRK2.7* and *HvSnRK3.32* were up-regulated at 3 h after ABA treatment. *HvSnRK3.23* was down-regulated at 6 h after ABA treatment, but not the other timepoints, while HvSnRK3.32 was down-regulated at 3 h after ABA treatment. *HvSnRK3.31* and *HvSnRK3.34* were up-regulated at 6 h after ABA treatment, while *HvSnRK3.30* down-regulated. Four genes (*HvSnRK3.12*, *HvSnRK3.21*, *HvSnRK3.22* and *HvSnRK3.28* were up-regulated only at 24 h after ABA treatment. Three further genes (*HvSnRK2.1*, *HvSnRK2.8* and *HvSnRK3.24*) showed more complicated responses, with *HvSnRK2.1* down-regulated at 1 h after ABA treatment, then up-regulated at 3 h, 6 h and 24 h, *HvSnRK2.8* up-regulated at 1 h after ABA treatment, then down-regulated at 6 h, and *HvSnRK3.24* down-regulated at 1 h after ABA treatment, then up-regulated at 3 h and 6 h. In all, half of the *HvSnRK2* genes and more than two thirds of the *HvSnRK3* genes responded to ABA, showing a complex picture of differential regulation. This is consistent with the *HvSnRK2* and *HvSnRK3* genes playing important roles in ABA responses [58, 59]. In contrast, of the 4 *HvSnRK1* genes that were expressed, none showed a significant change in expression in response to ABA. ABA has been shown to influence SnRK1 in wheat roots but at the post-translational rather than transcriptional level [47].

## Discussion

SnRKs play important roles in linking stress and ABA signalling with metabolic signalling in plants. Barley is the fourth most important cereal crop in the world in terms of production, and its widespread cultivation can be attributed in part to its good adaptation to different environments. This makes the elucidation of the *SnRK* gene family in this species particularly important. In this study, a total of 50 barley *HvSnRK* genes were identified, providing a basis for studying their roles individually, or as subfamilies or as an entire gene family.

BLAST searches or HMM searches or both are usually used for identifying gene homologues and families of homologues, and both types of search were adopted for this study. The methodology was validated by applying it to rice and Arabidopsis and showing that it identified all of the *SnRK* genes in those species. Halford and Hardie separated the SnRKs into three subgroups: SnRK1, SnRK2 and SnRK3, with the molecular weight of SnRK1 around 58 kDa and SnRK2 around 40 kDa, with more variability for SnRK3 [1]. The HvSnRKs separated clearly into these three subgroups, and clustered with the corresponding subgroups of rice and Arabidopsis in phylogenetic analyses, indicating that the three SnRK subgroups were established before the divergence of dicot and monocot plants. This was also inferred by Wang et al. [60] in the identification of BdSnRKs in *Brachypodium distachyon*. Motif analysis of the encoded proteins also distinguished the three subfamilies, and the presence/absence of different motifs could be used to assign SnRKs to the correct subfamily in species that are less well characterized and phylogenetic analysis is more difficult.

SnRK2s and SnRK3s are unique to plants, and have diverged further from SnRK1 than SnRK1 has from its fungal and animal counterparts, SNF1 and AMPK. Presumably the *SnRK2* and *SnRK3* genes first arose from duplications of the *SnRK1* gene, then evolved and diversified as they took on new roles, resulting in the burgeoning of the gene family as a whole. Our analysis revealed gene duplication (including segmental and tandem duplication) events in the evolution of the gene family, as well as differential regulation in response to ABA treatment. In addition, the Ka/Ks analysis indicated that the duplicated *HvSnRK* genes evolved slowly or were highly conserved [66].

*SnRK1* genes of cereals have been subdivided into *SnRK1a* and *SnRK1b* according to their expression patterns, with *SnRK1a* expressed in multiple tissues and *SnRK1b* expressed predominantly in the endosperm [1, 18, 19]. Curtis et al. [16] then identified a subclass similar to *SnRK1b* that was expressed in the embryo as well as the endosperm and named it SnRK1b*. These three subclasses of *HvSnRK1s* could be readily distinguished in the phylogenetic analysis in this study.

*SnRK2s* were also divided into two subclasses (a and b) by Halford and Hardie [1], then further into three subclasses (I, II and III) by Kobayashi et al. [33], with II/III corresponding to *SnRK2a* and I to *SnRK2b*. Subclass II has been reported to be strongly activated by ABA, Class II weakly activated and Class I not at all [41–44]. The regulation of the barley *HvSnRK2*s by ABA appears to be more complicated. Phylogenetic analysis of the barley gene family readily distinguished the *HvSnRK2a* Class II/III and HvSnRK1b Class I types; however, all but two of the *HvSnRK2s* (*HvSnRK2.8* and *HvSnRK2.10*) had ABA response elements in their promoters, and those that were shown to be significantly regulated by ABA treatment included members of all three subclasses.

Synteny analysis showed that one-to-one correspondence between the *SnRKs* of barley and rice was better than that between barley and Arabidopsis, consistent with the hypothesis of Wang et al. [60] that the monocot and dicot gene families have differentiated. The study also identified some *HvSnRKs* without corresponding genes in either rice or Arabidopsis, indicating continued divergence and differentiation of the barley gene family since its evolutionary line split from that of rice.

## Conclusions

The barley *HvSnRK* gene family comprises 50 *HvSnRK* genes, six of which are of the *HvSnRK1* subfamily, 10 *HvSnRK2* and 34 *HvSnRK3*. The presence/absence of specific motifs in the encoded proteins distinguishes between the subfamilies. Multiple putative regulatory elements are present in the gene promoters, with light-regulated elements (LRE), ABA response elements (ABRE) and methyl jasmonate response elements (MeJa) the most common. Many of the genes respond to ABA, some positively, some negatively and some with complex time-dependent responses.

## Methods

### Identification and phylogenetic analysis of the *SnRK* gene family in barley

The non-redundant amino acid sequences of Arabidopsis (*Arabidopsis thaliana*) and rice (*Oryza sativa*) SnRKs were collected from TAIR v10 (http://www.arabidopsis.org/) and RGAP v7 databases (http://rice.plantbiology.msu.edu/), respectively. Seventy-three SnRK sequences from Arabidopsis, 54 from rice and one (BKIN12) from barley were obtained directly from published papers [18, 36, 61, 62, 67] (Additional file 1). The entire predicted protein sequences of barley (*Hordeum vulgare*) were downloaded from the Ensembl Plants database (http://plants.ensembl.org/Hordeum_vulgare/Info/Index, IBSC v2). To identify candidate SnRKs in *Hordeum vulgare*, local Hidden Markov Model-based searches in the protein sequence dataset were performed separately with PF00069 HMM. In addition, BLAST searches with all the published SnRK sequences of Arabidopsis, rice and barley as queries were performed to identify the predicted SnRKs in the *Hordeum vulgare* protein database. All the potential HvSnRK proteins identified from the HMM and BLAST searches were validated for the presence of conserved domains with the NCBI CDD databases (http://www.ncbi.nlm.nih.gov/cdd/) [68] and SMART web tools (http://smart.embl.de/) [69]. The conserved protein kinase (Pkinase) domain regions of the SnRK family from Arabidopsis, rice and barley were then selected to perform multiple alignments using MAFFT v7.427 [70]; this region was selected and the C-terminal region omitted because of the high variation in the C-terminal sequences of the SnRK proteins. The amino acid substitution model was calculated by the ProTest-3.4.2 and the optimal model of “JTT + I + G + F” was selected [71]. The RAxML v8.2.9 program was used to construct the maximum-likelihood (ML) tree, with bootstrap values for 1000 replicates [72], and FigTree was used to draw the rooted tree.

### Protein properties and sequence analyses

The molecular weights and isoelectric points of putative HvSnRK proteins were calculated by the ExPASy proteomics server (http://expasy.org/). MEGA 7.0 was used for constructing an unrooted phylogenetic tree for the HvSnRK family proteins. In brief, all of the barley SnRK protein sequences were aligned using ClustalW with the default parameters, and the phylogenetic tree was built using the Neighbor-Joining (NJ) method within MEGA 7.0, with 1000 bootstrap replications. The MEME program (http://meme.sdsc.edu/meme/intro.html) was used to identify the motifs, and information on the *HvSnRK* genes was obtained from the *Hordeum vulgare* genome database (Ensembl Plants). The gene structures and protein motifs were drawn by TB tools [73]. To analyze the *cis*-elements in the promoter regions, the 1.5 kb upstream region of the coding sequence region of each *HvSnRK* gene (Additional file 1) was analyzed with the PlantCARE databases [74] (http://bioinformatics.psb.ugent.be/webtools/plantcare/html/), and the *cis*-elements were drawn by TB tools.

### Chromosomal location, genome synteny and gene duplication analyses

The chromosomal location of the *HvSnRK* genes was downloaded from the *Hordeum vulgare* Genome Database (Ensembl Plants), and the distribution of the genes on chromosomes was drawn using the MG2C v.2 program (http://mg2c.iask.in/mg2c_v2.0/). Gene duplication, including segmental and tandem duplication, was analyzed using the MCScanX program [75]. Genes were considered to be segmentally duplicated if they occurred in collinear segments containing at least five collinear gene pairs, whereas they were considered to be tandemly duplicated if they were located close to each other with no more than two interval genes. The KaKs ratio (the ratio of nonsynonymous (Ka) to synonymous (Ks) nucleotide substitution rates) was also calculated for the duplicated gene pairs using the KaKs Calculator 2.0 [76].

### Plant materials and ABA treatments

Barley seeds of variety Morex (It was original from Jiangsu Coastal Area Institute of Agricultural Sciences, Jiangsu, China, and maintained at the Biotechnology Research Institute of Shanghai Academy of Agricultural Sciences by Chenghong Liu and Zhiwei Chen.) were sterilized with 1% NaClO for 30 min, and germinated in an incubator at 25 °C for 4 days. Seedlings were transferred into plastic boxes within foam boards in them and cultured in nutrient solution mainly according to Chen et al. [73]. At the two to three-leaf stage, half of the seedlings were treated with 50 μM ABA dissolved in ethanol and the other half were treated with ethanol alone. Roots were harvested separately at 1 h, 3 h, 6 h and 24 h after treatment, and samples were frozen in liquid nitrogen and keep at − 80 °C until required. There were three biological replicates for each sample.

### cDNA library construction and RNA-sequencing

Total RNA isolation and quality control were carried out according to Chen et al. [73], and 1.5 μg total RNA per sample was used for RNA preparations. The NEBNext® Ultra™ Directional RNA Library Prep Kit for Illumina (NEB, USA) was used to generate sequencing libraries, following the manufacturer’s instructions, and mRNA was purified from total RNA using poly-Toligo-attached magnetic beads. Nucleotide sequence analysis of the libraries was then carried out on an Illumina Hiseq Xten platform (Illumina Inc., San Diego, CA), and 150 bp paired-end reads were generated. The clean nucleotide sequence data ranged from 7.95 to 15.06 Gb (all > 6 Gb), and the Q30 percentages were all > 80% (Table S1). These results suggested that the data were sufficient and reliable enough for further analysis. Spearman correlation analysis also showed that the three biological replicates of each sample met the requirements (all over 0.95) (Figure S1).

### Expression analysis of *HvSnRKs* by RNA-seq

Reference genome and gene model annotation files were downloaded directly from the Ensembl plants database (http://plants.ensembl.org/Hordeum_vulgare/Info/Index, IBSC v2). An index of the reference genome was built and clean reads aligned to the reference genome using Hisat2 v2.0.5. HTSeq v0.6.1 was used to count the read numbers that mapped to each gene. The number of reads per kilobase of transcript, per million mapped reads (RPKM) was calculated based on the length of the gene and the number of reads that mapped to the gene. Differential expression analysis of the two treatments (with or without ABA) was performed using the DESeq2 R package (1.16.1). Genes with a *p* value < 0.05 were assigned as significantly differentially-expressed

## Supplementary Information

**Additional file 1. **SnRK peptides used for the blast, *SnRK* genes in Arabidopsis, rice and barley, and 1500 bp upstream nucleic acids sequences of *HvSnRKs*.

**Additional file 2. **Promoter predictions of *HvSnRKs*.

**Additional file 3.** Synteny analysis between Arabidopsis/rice and barley.

**Additional file 4. **Up/down-regulated *HvSnRKs* genes response to ABA treatment.

**Additional file 5: Figure S1.** Spearman correlations of gene expressions among all samples.

**Additional file 6: Figure S2.** Amino acid sequences of the ten motifs for HvSnRK proteins. The larger the letters represent the higher ratio of the amino acid at each site.

**Additional file 7: Table S1.** Summary of RNA-seq data from barley roots treated with or without ABA at different time points (three biological replicates for each sample).

## Data Availability

The genome and gene annotation file of Arabidopsis (*Arabidopsis thaliana*), rice (*Oryza sativa*) SnRKs and barley (*Hordeum vulgare*) were collected from TAIR v10 (Peptide data: https://www.arabidopsis.org/download_files/Sequences/TAIR10_blastsets/TAIR10_pep_20101214_updated; Annotation data: https://www.arabidopsis.org/download_files/Genes/TAIR10_genome_release/TAIR10_gff3/TAIR10_GFF3_genes.gff; Genome data: https://www.arabidopsis.org/download_files/Genes/TAIR10_genome_release/TAIR10_chromosome_files/TAIR10_chr_all.fas .), RGAP v7 databases (Peptide data: http://rice.plantbiology.msu.edu/pub/data/Eukaryotic_Projects/o_sativa/annotation_dbs/pseudomolecules/version_7.0/all.dir/all.pep; Annotation data: http://rice.plantbiology.msu.edu/pub/data/Eukaryotic_Projects/o_sativa/annotation_dbs/pseudomolecules/version_7.0/all.dir/all.gff3; Genome data: http://rice.plantbiology.msu.edu/pub/data/Eukaryotic_Projects/o_sativa/annotation_dbs/pseudomolecules/version_7.0/all.dir/all.con .) and the Ensembl Plants database (Peptide data: ftp://ftp.ensemblgenomes.org/pub/plants/release-50/fasta/hordeum_vulgare/pep/Hordeum_vulgare.IBSC_v2.pep.all.fa.gz; Annotation data: ftp://ftp.ensemblgenomes.org/pub/plants/release-50/gtf/hordeum_vulgare/Hordeum_vulgare.IBSC_v2.50.gtf.gz; Genome data: ftp://ftp.ensemblgenomes.org/pub/plants/release-50/fasta/hordeum_vulgare/dna/Hordeum_vulgare.IBSC_v2.dna_sm.toplevel.fa.gz .), respectively. The RNA-seq data have been deposited with the National Center for Biotechnology Information: BioProject ID PRJNA661163.

## Abbreviations

SnRK: Sucrose nonfermenting-1-related protein kinase
ABA: Abscisic acid
LRE: Light-regulated elements
ABRE: ABA response elements
MeJa: Methyl jasmonate response elements
RNA-seq: RNA sequencing
SNF1: Sucrose non-fermenting 1
AMPK: AMP-activated protein kinase
SIP: SNF1 interacting protein
HMG-CoA: 3-hydroxy-3-methylglutaryl coenzyme A
ADP: Adenosine diphosphate
UDP: Uridine diphosphate
DON: Deoxynivalenol
VIGS: Virus-induced gene silencing
AREBP: ABA-response-element-binding protein
CBL: Calcineurin B-like protein
CIPK: CBL-interacting protein kinase
SOS: Salt overly sensitive
