# Supplementary figures and images for: Genome-wide identification of sucrose nonfermenting-1-related protein kinase (SnRK) genes in barley and RNA-seq analyses of their expression in response to abscisic acid treatment
Source: BMC Genomics. 2021 Apr 26;22:300. doi: 10.1186/s12864-021-07601-6 (PMC8074225; doi:10.1186/s12864-021-07601-6)

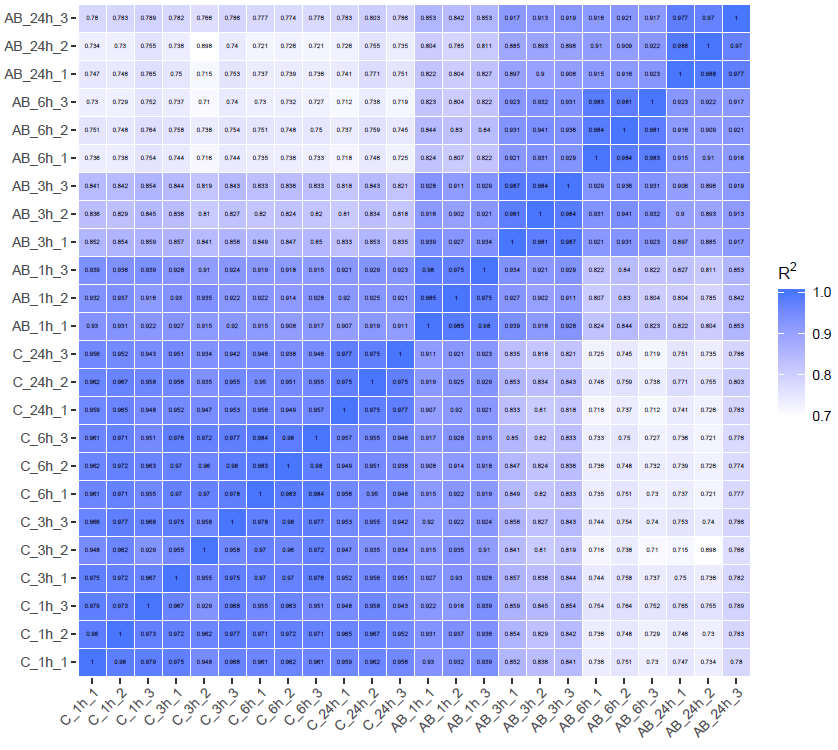


Figure S1. Spearman correlations of gene expressions among all samples

Supplement: Supplementary file 5 — Additional file 5: Figure S1. Spearman correlations of gene expressions among all samples. [file 12864_2021_7601_MOESM5_ESM.docx]
