# Supplementary material for: Genome-wide identification of sucrose nonfermenting-1-related protein kinase (SnRK) genes in barley and RNA-seq analyses of their expression in response to abscisic acid treatment
Source: BMC Genomics. 2021 Apr 26;22:300. doi: 10.1186/s12864-021-07601-6 (PMC8074225; doi:10.1186/s12864-021-07601-6)
Supplement: Supplementary file 6 — Additional file 6: Figure S2. Amino acid sequences of the ten motifs for HvSnRK proteins. The larger the letters represent the higher ratio of the amino acid at each site. [file 12864_2021_7601_MOESM6_ESM.docx]

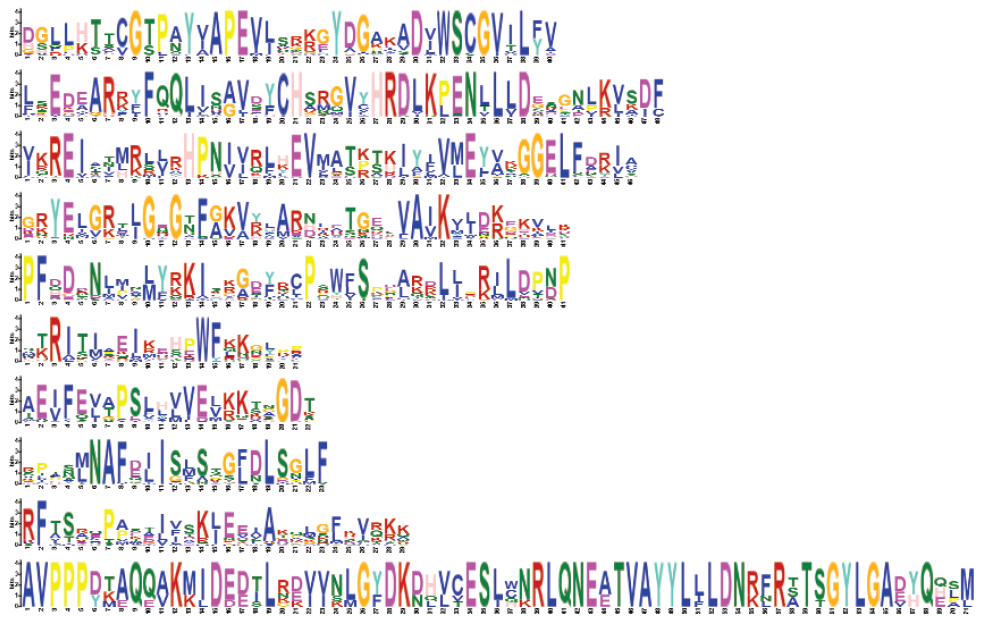


Motif 1

Motif 2

Motif 3

Motif 4

Motif 6

Motif 9

Motif 5

Motif 8

Motif 7

Motif 10

Figure S2. Amino acid sequences of the ten motifs for HvSnRK proteins. The larger the letters represent the higher ratio of the amino acid at each site.
