# Supplementary material for: Genome-wide identification of sucrose nonfermenting-1-related protein kinase (SnRK) genes in barley and RNA-seq analyses of their expression in response to abscisic acid treatment
Source: BMC Genomics. 2021 Apr 26;22:300. doi: 10.1186/s12864-021-07601-6 (PMC8074225; doi:10.1186/s12864-021-07601-6)
Supplement: Supplementary file 7 — Additional file 7: Table S1. Summary of RNA-seq data from barley roots treated with or without ABA at different time points (three biological replicates for each sample). [file 12864_2021_7601_MOESM7_ESM.docx]

Table S1 Summary of RNA-seq data from barley roots treated with or without ABA at different time points (three biological replicates for each sample)

|  |  |  | Raw bases (Gb) | Clean bases (Gb) | Clean Q30 (%) | Clean GC (%) |
| --- | --- | --- | --- | --- | --- | --- |
| CK | 1h | 1st | 10.06 | 9.89 | 92.21 | 54.89 |
|  |  | 2nd | 8.81 | 8.68 | 92.57 | 54.92 |
|  |  | 3rd | 10.74 | 10.56 | 92.38 | 54.36 |
|  | 3h | 1st | 9.37 | 9.22 | 92.59 | 55.33 |
|  |  | 2nd | 9.01 | 8.87 | 92.63 | 55.09 |
|  |  | 3rd | 9.43 | 9.28 | 92.69 | 55.21 |
|  | 6h | 1st | 10.48 | 10.30 | 92.39 | 54.91 |
|  |  | 2nd | 8.81 | 8.66 | 92.49 | 54.62 |
|  |  | 3rd | 8.95 | 8.83 | 92.52 | 55.02 |
|  | 24h | 1st | 8.06 | 7.95 | 92.73 | 54.79 |
|  |  | 2nd | 9.49 | 9.36 | 92.77 | 54.60 |
|  |  | 3rd | 10.86 | 10.61 | 89.94 | 55.66 |
| ABA treatment | 1h | 1st | 10.86 | 10.56 | 90.05 | 57.06 |
|  |  | 2nd | 11.88 | 11.59 | 89.71 | 56.40 |
|  |  | 3rd | 12.27 | 11.98 | 89.74 | 56.75 |
|  | 3h | 1st | 10.38 | 10.11 | 89.89 | 56.05 |
|  |  | 2nd | 10.52 | 10.26 | 90.10 | 56.40 |
|  |  | 3rd | 9.10 | 8.85 | 89.79 | 56.33 |
|  | 6h | 1st | 11.26 | 10.96 | 89.64 | 56.63 |
|  |  | 2nd | 10.31 | 10.03 | 89.62 | 57.31 |
|  |  | 3rd | 14.80 | 14.05 | 84.18 | 57.24 |
|  | 24h | 1st | 14.49 | 13.78 | 84.07 | 56.36 |
|  |  | 2nd | 15.83 | 15.06 | 83.76 | 56.11 |
|  |  | 3rd | 11.69 | 11.44 | 93.40 | 55.32 |
